# Ixazomib-based frontline therapy in patients with newly diagnosed multiple myeloma in real-life practice showed comparable efficacy and safety profile with those reported in clinical trial: a multi-center study

**DOI:** 10.1007/s00277-020-04234-9

**Published:** 2020-09-06

**Authors:** Jing Li, Li Bao, Zhongjun Xia, Sili Wang, Xin Zhou, Kaiyang Ding, Wenhao Zhang, Wei Yang, Bingzong Li, Chengcheng Fu, Bing Chen, Luoming Hua, Liang Wang, Jun Luo, Yang Yang, Tianhong Xu, Weida Wang, Yun Huang, Guolin Wu, Peng Liu

**Affiliations:** 1 Department of Hematology, Zhongshan Hospital, Fudan University, 180 Fenglin Road, Shanghai, 200032 People’s Republic of China; 2 Department of Hematology, Beijing Jishuitan Hospital, Beijing, China; 3 Department of Hematologic Oncology, Sun Yat-sen University Cancer Center, Guangzhou, China; 4 The First Affiliated Hospital of Xiamen University, Xiamen, China; 5 Wuxi People Hospital, WuXi, China; 6 Anhui Provincial Cancer Hospital, Hefei, China; 7 Xinhua Hospital Affiliated to Shanghai Jiaotong University School of Medicine, Shanghai, China; 8 Shengjing Hospital of China Medical University, Shengjing, China; 9 The Second Affiliated Hospital of Soochow University, Suzhou, Jiangsu China; 10 Department of Hematology, The First Affiliated Hospital of Soochow University, Jiangsu Institute of Hematology, Suzhou, China; 11 Department of Hematology, The Affiliated Nanjing Drum Tower Hospital, Nanjing University Medical School, Nanjing, China; 12 The Affiliated Hospital of Hebei University, Baoding, China; 13 Department of Hematology, ZhuJiang Hospital of Southern Medical Univeristy, Guangzhou, China; 14 The First Affiliated Hospital of Guangxi Medical University, Nanning, Guangxi China

**Keywords:** Myeloma, Frontline, Ixazomib, Real-world

## Abstract

The induction therapy containing ixazomib, an oral proteasome inhibitor, has shown favorable efficacy and safety in clinical trials, but its experience in real-life remains limited. In routine practice, few patients received ixazomib-based induction therapy due to reasons including (1) patients’ preference on oral regimens, (2) concerns on adverse events (AEs) of other intravenous/subcutaneous regimens, (3) requirements for less center visits, and (4) fears of COVID-19 and other infectious disease exposures. With the aim of assessing the real-life effectiveness and safety of ixazomib-based induction therapy, we performed this multi-center, observational study on 85 newly diagnosed multiple myeloma (NDMM) patients from 14 medical centers. Ixazomib-based regimens included ixazomib-lenalidomide-dexamethasone (IRd) in 44.7% of patients, ixazomib-dexamethasone (Id) in 29.4%, and Id plus another agent (doxorubicin, cyclophosphamide, thalidomide, or daratumumab) in 25.9%. Different ixazomib-based therapies were applied due to (1) financial burdens or limitations on local health insurance coverage, (2) concerns on treatment tolerance, and (3) drug accessibility issue. Ten patients received ixazomib maintenance. The median age was 67 years; 43.5% had ISS stage III disease; 48.2% had an Eastern Cooperative Oncology Group performance score ≥ 2; and 17.6% with high-risk cytogenetic abnormalities. Overall response rate for all 85 patients was 95.3%, including 65.9% very good partial response or better and 29.5% complete responses. The median time to response was 30 days. The response rate was similar across different ixazomib-based regimens. Median progression-free survival was not reached. Severe AEs (≥ grade 3) were reported in 29.4% of patients. No grade 3/4 peripheral neuropathy (PN) occurred. Patients received a median of 6 (range 1–20) cycles of ixazomib treatment; 56.6% remained on treatment at data cutoff; 15.3% discontinued treatment due to intolerable AEs. These results support that the ixazomib-based frontline therapy was highly effective with acceptable toxicity in routine practice and the ixazomib oral regimens could be good alternative options for NDMM patients.

## Introduction

Substantial improvement in outcomes of multiple myeloma (MM) has been observed over the past decades [1], and the treatment paradigm for MM has been considerably evolved [2]. For newly diagnosed MM (NDMM), induction therapy with multi-agents combination has become a standard of care, with numeral phase 3 trials showing superior survival benefit using triplet or quadruplet regimens containing proteasome inhibitor bortezomib [3–5]. Besides, the concept of continuous or long-term therapy in MM has been widely accepted, since maintenance therapy or continued initial therapy demonstrated obvious prolonged survival [6, 7]. In this context, the proteasome inhibitor bortezomib has become one of the backbone agents in the frontline treatment for MM; however, the non-oral administration and concerns in side effects including peripheral neuropathy hampered its long-term use in the real-world setting, with a median duration of treatment of only 6 months for bortezomib-based frontline therapy [8].

Ixazomib is the first oral proteasome inhibitor approved for the treatment in MM patients who have received at least one prior therapy in over 60 countries. The all-oral combination of weekly ixazomib plus lenalidomide and dexamethasone (IRd) has demonstrated durable efficacy and well-tolerated toxicities in phase 3 trial TOURMALINE-MM1 [9, 10] and in real-life practice [11].

The all-oral triplet regimen of IRd exhibited favorable efficacy with an acceptable toxicity profile in NDMM, according to the results of a phase 1/2 study (NCT01217957) [12, 13], with an overall response rate (ORR) of 92%. Besides, the ixazomib-based all-oral regimen combined with cyclophosphamide and dexamethasone (ICd) has shown promising efficacy in phase 2 studies [14, 15]. Furthermore, all-oral regimens, including IRd combination, have been suggested as possible options during the pandemic of COVID-19 in different consensus or opinions [16–19], owing to the fact that using an oral regimen may help to reduce the risk for virus exposure and infection. However, effectiveness and tolerability of novel agent for MM treatment in routine clinical practice often differ from the results reported in registered clinical trials [8, 20, 21], and the published data on frontline treatment with ixazomib-based regimes in large phase 3 trials and in the real-world setting are limited. Herein, we report the real-world data of the initial effectiveness and safety profile of ixazomib-based frontline therapy in patients with NDMM from 14 centers in China, in hopes of providing informative data to help therapeutic decision-making.

## Patients and methods

This is a national, multi-centric, retrospective, observational study analyzing data collected from 85 patients with NDMM, who received ixazomib-containing regimen as induction therapy in real-life routine practice. Patients were treated at 14 different medical centers in China between August 2018 and April 2020, since ixazomib was officially approved by the China Food and Drug Administration in April 2018.

For inclusion in the present study, patients had to be diagnosed with MM by the 2014 criteria of International Myeloma Working Group (IMWG) [22] and previously untreated before receiving at least one cycle of ixazomib therapy. The records including demographics, disease characteristics, molecular cytogenetic subtype, treatment exposure, treatment response, treatment duration, adverse events (AEs), and survival outcome were retrieved and analyzed.

The main objectives of this analysis were to determine the ORR (partial response (PR) or better) and the proportion of patients with CR or very good PR (VGPR) of frontline ixazomib-based regimens in real-life setting. Secondary endpoints included estimation of progression-free survival (PFS), overall survival (OS), and the response rate within patient subgroups categorized by cytogenetic risk stratification defined by fluorescence in situ hybridization (FISH) [23] or metaphase cytogenetics. High-risk cytogenetic abnormalities (CA) included del 17, *t*(4;14), *t*(14;16).

The other primary objective of this study was to assess the safety profile of frontline ixazomib therapy, including the occurrence rate of common AEs and severe AEs, the duration of ixazomib treatment exposure, and the rate and reasons for drug discontinuation.

The study was approved by the institutional ethics committee (B2019-228R) and conducted in accordance with the 1964 Helsinki declaration and its later amendments. Patient care and evaluation were determined by their own treating physicians and effected by the patients’ own preference.

All 85 patients with symptomatic MM received at least one ixazomib-based therapy. Ixazomib-containing regimens analyzed in this study were listed as follows: IRd regimen (ixazomib 4 mg on days 1, 8, and 15, lenalidomide 25 mg on days 1–21, dexamethasone 40 mg weekly, every 28 days), Id regimen (ixazomib 4 mg on days 1, 8, and 15, dexamethasone 40 mg weekly, every 28 days), and Id regimen in combination with other chemotherapeutic agents or monoclonal antibody (ITd, Id plus daily thalidomide 100 mg; ICd, Id plus weekly oral cyclophosphamide 300 mg/m^2^; IAd, Id plus doxorubicin 9 mg/m^2^ IV d1–4; DId, Id plus daratumumab administered IV at 16 mg/kg weekly for 8 weeks, followed by every other week for 8 doses, and then every 4 weeks). Selected patients received long-term single-agent ixazomib maintenance after up to 12 cycles of ixazomib-based induction therapy. Patients received continuous ixazomib-based treatment until disease progression, intolerable toxicity, or patient/physician decision to end treatment. Dose modifications of studying drugs for toxic effects were recorded. Interruption of treatment for stem cell collection or stem cell transplantation (SCT) was allowed.

Response assessment and safety evaluation were done in patients every month according to the 2016 IMWG response criteria [24]. A bone marrow aspiration/biopsy was required to confirm a complete response (CR).

Statistical analysis was conducted using SPSS statistics software (version 26, IBM, Armonk, NY). Statistical significance was set at a two-sided value of *P* < 0.05. Descriptive statistics were used to describe baseline characteristics, response, and AEs data. The Chi-square method or Fisher’s exact test was used to determine differences between nominal variables. Survival curves were calculated using the Kaplan-Meier curves, and log-rank tests were performed to assess the differences between the groups.

## Results

### Patients

A total of 85 patients with NDMM from 14 medical centers treated with at least one cycle of ixazomib-based regimens as frontline induction therapy were included in this study. Table 1 summarized the patient demographic and disease features at the time of diagnosis. The overall median age at diagnosis was 67 years old (range, 35–87). Almost half (43.5%) of patients had an advanced ISS stage III disease, and 48.2% of patients had an Eastern Cooperative Oncology Group (ECOG) performance score of ≥ 2. On cytogenetics and FISH analysis (data available in 68 patients), 17.6% of patients were identified with high-risk CA, including 4 (5.9%) patients with del 17p, 11 (16.2%) with *t*(4;14), and 4 (5.9%) with *t*(14;16). Extramedullary lesion was found in 6 (7.1%) patients. On the basis of exclusion criteria of the phase 1/2 study (NCT01217957) evaluating IRd regimen in NDMM [12], 30 (35.3%) patients were ineligible for this clinical trial. Criteria leading to ineligibility in the clinical trial included ECOG PS of more than 2 (70.0%), grade 2 or higher peripheral neuropathies (10.0%), uncontrolled cardiovascular conditions within the past 6 months (10.0%), prior malignancy within 2 years (3.3%), and others (6.7%).

**Table 1:** Baseline clinical characteristics of the patients included in the analysis

Characteristic	All Patients (*N* = 85)
Median age (range)	67 (35–87)
Age group (%)	
18–64 years	41 (48.2)
65–74 years	26 (30.6)
≥ 75 years	18 (21.1)
Male, *n* (%)	48 (56.5)
ISS stage at diagnosis, *n* (%)	
I	22 (25.9)
II	26 (30.6)
III	37 (43.5)
High-risk cytogenetic abnormalities^a,b^ (%)	15 (17.6)
Del 17	4 (5.9)
*t*(4;14)	11 (16.2)
*t*(14;16)	4 (5.9)
ECOG performance status, *n* (%)	
0–1	44 (51.8)
2	20 (23.5)
3–4	21 (24.7)
Serum creatinine > 177 μmol/L, *n* (%)	18 (21.2)
Extramedullary disease	6 (7.1)
Eligibility for phase I/II trial^c^	30 (35.3)
Regimens, *n* (%)	
Ixazomib plus lenalidomide-dexamethasone (IRd)	38 (44.7)
Ixazomib plus dexamethasone (Id)	25 (29.4)
Id plus chemotherapeutics/monoclonal antibody^d^	22 (25.9)

*ISS*, International Staging System

^a^High-risk cytogenetic abnormalities were detected by fluorescence in situ hybridization (FISH) or metaphase cytogenetics, including del 17, *t*(4;14), *t*(14;16)

^b^17 patients with no FISH data were not included

^c^Patients whose baseline characteristics theoretically fulfilled the in- and exclusion criteria for the phase 1/2 study (NCT01217957, a study evaluated the safety and efficacy of IRd regimen, followed by single-agent ixazomib maintenance, in patients with newly diagnosed myeloma) were defined as eligible for phase I/II trial

^d^This subgroup included Id plus other chemotherapeutics or monoclonal antibody: 11 cases with doxorubicin, 7 with cyclophosphamide, 3 with thalidomide, and 1 with daratumumab

### Treatment regimen and exposure

Ixazomib-based regimens given to all patients were shown in Table 1, including IRd regimen in 38 (44.7%) patients, Id in 25 (29.4%) patients, and Id plus chemotherapeutics/other agents (11 cases with doxorubicin, 7 with cyclophosphamide, 3 with thalidomide, and 1 with daratumumab) in 22 (25.9%). A total of 10 patients received single-agent ixazomib maintenance after median 8 (range, 6–12) cycles of ixazomib-containing induction therapy (Table 2). Six (7.1%) of the patients received SCT after induction therapy with ixazomib.

**Table 2:** Treatment exposure in all patients and patients who received maintenance

	All patients (*N* = 85)	Patients who received ixazomib maintenance (*N* = 10)
Median cycles of ixazomib received, *n* (range)	6 (1–20)	14 (11–20)
Cycles of ixazomib received, *n* (%)		
≥ 3	74 (87.1)	10 (100.0)
≥ 6	51 (60.0)	10 (100.0)
≥ 9	24 (28.2)	10 (100.0)
Number of patients with dose reduction, *n* (%)	3 (3.6)	0
Patients proceeded to SCT	6 (7.1)	0
Patients remaining on treatment, *n* (%)	48 (56.5)	9 (90.0)
Reason for ending treatment, *n* (%)		
Adverse event	13 (15.3)	0
Disease progression	6 (7.1)	1 (10.0)
Another ^a^	18 (21.1)	0

*SCT*, stem cell transplantation

^a^Reasons included proceeding to SCT, alternate therapy, poor compliance, loss of drug accessibility during the pandemic of COVID-19 and economic concerns

Treatment exposure for all patients and those who received ixazomib maintenance were documented in Table 2. At data cutoff, the median follow-up time for all patients included was 10.3 months (range, 1.1–22.9). During the follow-up period, the median cycles of ixazomib therapy received by all patients and those who had ixazomib maintenance were 6 (range, 1–20) cycles and 14 (range, 11–20), respectively. At data cutoff, 56.5% (48/85) of patients remained on ixazomib treatment, including 90% (9/10) of those receiving single-agent ixazomib as maintenance. For the remaining 43.5% of patients who discontinued treatment, the reason for treatment discontinuation (patients could have more than one reason listed) included AEs in 13 (15.3%) patients, disease progression in 6 (7.1%), and other specific reasons in 18 (21.1%). Other reasons included proceeding to SCT, alternate therapy, poor compliance, economic pressure, and loss of drug accessibility during the pandemic of COVID-19.

### Response and outcome

**Fig. 1:**
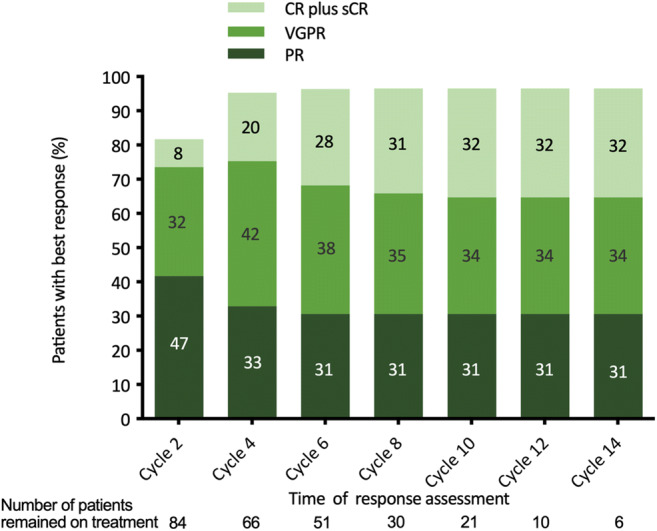
Changes in response with increasing duration of therapy

The best confirmed ORR for all 85 patients was 95.3% (81/85), including 65.9% of patients with ≥ VGPR and 29.5% with a CR (including stringent CR) (details are shown in Table 3). The median time to 1st documented response was 30 days, and the median time to best response was 64 days. The kinetics of response during follow-up are shown in Fig. 1, and the response deepened with time. Among the 15 data-evaluable patients who had high-risk CA, the ORR was 86.7%, including a 33.3% ≥ VGPR rate and a 13.3% CR rate. Although the ORR for high-risk CA subgroup was comparable with the overall population, the CR rate and ≥ VGPR rate were much lower (*p* = 0.016). There was no significant difference in best confirmed response and time to response among patients who received different ixazomib-based regimens, as shown in Table 3.

**Table 3:** Treatment outcomes in all patients, those who had high-risk CA, and those who received different ixazomib-based regimens

	All patients (*N* = 85)	High-risk CA^a^ (*n* = 15)	IRd regimen (*n* = 38)	Other triplet regimen^b^ (*n* = 22)	Id doublet regimen (*n* = 25)
Best confirmed response^c^, *n* (%)					
ORR (≥ PR)	81 (95.3)	13 (86.7)	35 (92.1)	21 (95.5)	25 (100.0)
≥ VGPR	56 (65.9)	5 (33.3)	22 (57.9)	19 (86.3)	15 (60.0)
CR	25 (29.5)	2 (13.3)	10 (26.3)	7 (31.8)	8 (32.0)
PR	56 (65.9)	11 (73.3)	25 (65.8)	14 (63.6)	17 (68.0)
VGPR	31 (36.5)	3 (20.0)	12 (31.6)	12 (54.5)	7 (28.0)
MR	1 (1.2)	1 (6.7)	1 (2.6)	0	0
SD	3 (3.5)	1 (6.7)	2 (5.3)	1 (4.5)	0
Median time to 1st response, months	1.0	1.0	1.0	1.0	0.9
Median time to best response, months	2.1	1.9	2.3	2.5	1.8
Median PFS, months	NE	NE	NE	NE	NE
12-month PFS, %	86.3	76.6	85.5	94.7	83.8
Median OS, months	NE	NE	NE	NE	NE
12-month OS, %	95.3	100	100	95.6	89.7
24-month OS, %	84.3	66.7	66.7	95.6	89.7
Patients with progression, *n* (%)	11 (12.9)	4 (26.7)	6 (15.8)	1 (4.5)	4 (16.0)

*CA*, cytogenetic abnormalities; *ORR*, overall response rate; *PR*, partial response; *VGPR*, very good partial response; *CR*, complete response; *MR*, minimal response; *SD*, stable disease; *PFS*, progression-free survival; *OS*, overall survival; *IRd*, ixazomib plus lenalidomide and dexamethasone; *Id*, ixazomib plus dexamethasone; *NE*, not estimable

^a^High-risk CA included del 17, *t*(4;14), *t*(14;16)

^b^This subgroup included Id plus another chemotherapeutics or monoclonal antibody: 11 cases with doxorubicin, 7 with cyclophosphamide, 3 with thalidomide, and 1 with daratumumab

^c^For patients who proceeded to SCT and received further ixazomib maintenance therapy, the best response reported include response post SCT

**Fig. 2:**
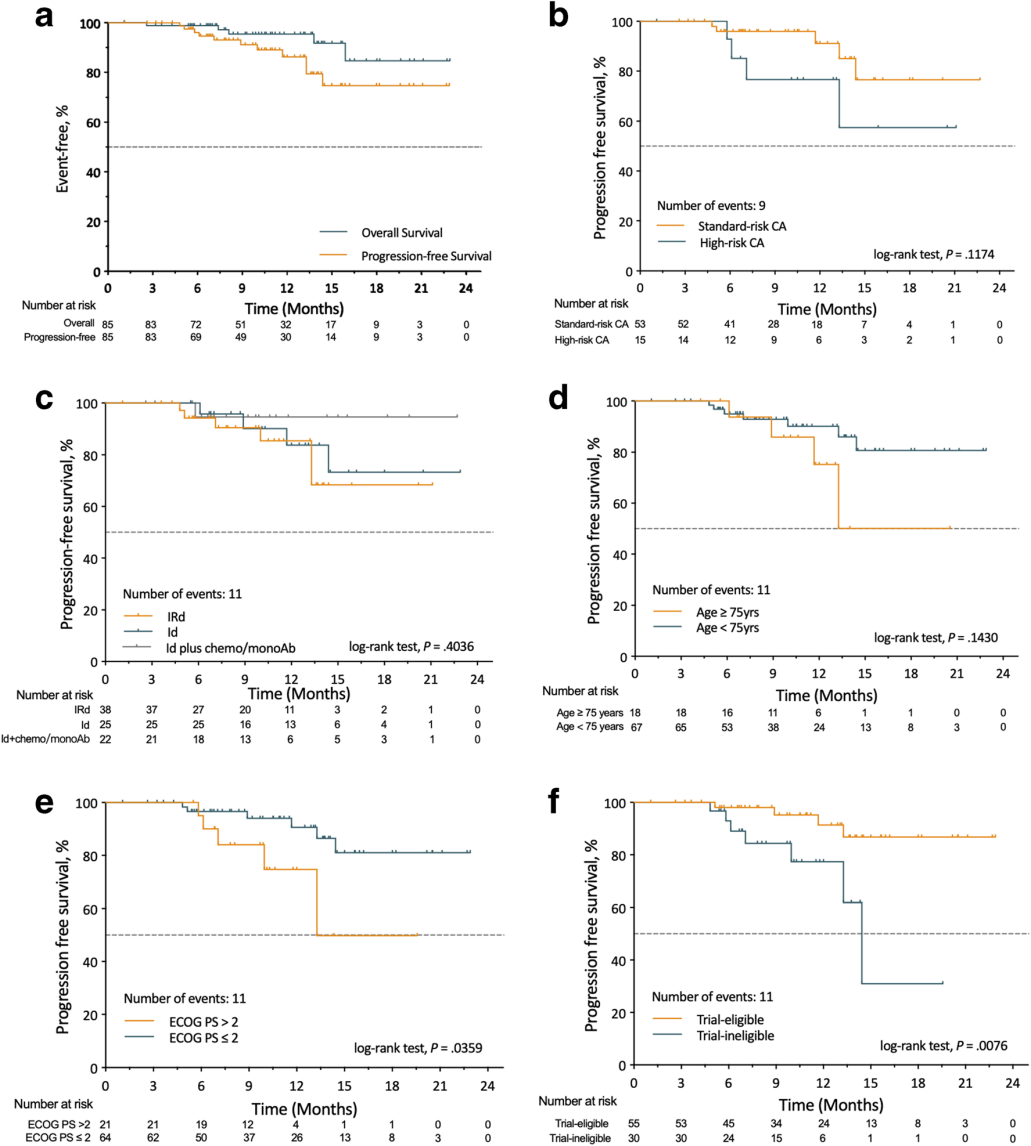
Kaplan-Meier survival curves. **a** Overall survival and progression-free survival (PFS) in all patients; PFS according to risk stratification by cytogenetic abnormalities (**b**), different ixazomib based regimens (**c**), age (**d**), ECOG performance status (**e**), and trial eligibility (**f**) based on the criteria of a phase 1/2 study evaluated the weekly oral ixazomib combined with Rd in patients with newly diagnosed myeloma (NCT01217957)

OS and PFS for the entire cohort are shown in Fig. 2A. Median OS and median PFS are not estimable (NE) in the whole population and any of the subgroups. At data cutoff, a total of 11 (12.9%) patients experienced disease progression. On landmark analysis, the 12-month PFS and OS for the entire cohort were 86.3% and 95.3%, respectively. No statistically significant difference was found among patients with different risk stratification in CA (Fig. 2B), those who received different ixazomib-based regimens (Fig. 2C), and patients who were at age of more than 75 years old (Fig. 2D). However, patients who had an ECOG PS > 2 (Fig. 2E) and those who were not eligible for the phase 1/2 trial of IRd in NDMM (NCT01217957) according to its inclusion/exclusion criteria (Fig. 2F) demonstrated inferior PFS (*p* < 0.05) in subgroup analysis.

### Safety profile

Table 4 summarizes the safety profile and common AEs of all patients and those who received different ixazomib-based regimen. Severe adverse events (≥ grade 3) were reported in 29.4% patients, including thrombocytopenia (7 patients, 8.2%), lymphocytopenia (3 patients, 3.5%), diarrhea (5 patients, 5.9%), pneumonia (6 patients, 7.0%), hypokalemia (1 patient, 1.7%), and severe skin/subcutaneous infection (2 patients, 2.4%). Peripheral neuropathy (PN) was reported in 5 patients, but there was no grade 3/4 peripheral neuropathy (PN) occurred. However, we believed some cases with mild PN might have been miss-recorded due to the retrospective nature of this analysis.

**Table 4:** Safety profile and common AEs of all patients and those who received different ixazomib-based regimen

	All patients (*N* = 85)	IRd regimen (*n* = 38)	Other triplet regimen^a^ (*n* = 22)	Id doublet regimen (*n* = 25)
Overall safety profile, *n* (%)				
Any grade ≥ 3 AE	25 (29.4)	9 (23.7)	7 (31.8)	9 (36.0)
AE leading to discontinuation of ixazomib	12 (14.1)	5 (13.2)	3 (13.6)	4 (16.0)
AE leading to dose reduction of ixazomib	3 (3.5)	1 (2.6)	2 (9.1)	0
Common hematologic AEs, *n* (%)				
Neutropenia	24 (28.2)	11 (28.9)	8 (36.4)	5 (20.0)
Thrombocytopenia	24 (28.2)	7 (18.4)	8 (36.4)	9 (36.0)
Anemia	27 (31.8)	12 (31.6)	8 (36.4)	10 (40.0)
Lymphocytopenia	29 (34.1)	14 (36.8)	5 (22.7)	12 (31.6)
Common non-hematologic AEs, *n* (%)				
Diarrhea	16 (18.8)	4 (10.5)	3 (13.6)	9 (36.0)
Nausea	8 (9.4)	0	5 (22.7)	3 (12.0)
Vomiting	8 (9.4)	0	5 (22.7)	3 (12.0)
Constipation	15 (17.6)	8 (21.1)	2 (9.1)	5 (20.0)
Fatigue	25 (29.4)	11 (28.9)	5 (22.7)	9 (36.0)
Upper respiratory tract infection	3 (3.5)	1 (2.6)	0	2 (8.0)
Rashes or other skin and subcutaneous tissue disorder except herpes zoster	20 (23.5)	16 (42.1)	1 (4.5)	3 (12.0)
Herpes zoster	2 (2.4)	0	0	2 (8.0)
Peripheral edema	11 (12.9)	4 (10.5)	1 (4.5)	6 (24.0)
Pneumonia	10 (11.8)	5 (13.2)	2 (9.1)	3 (12.0)
Peripheral neuropathy	5 (5.9)	1 (2.6)	0	4 (16.0)

*AE*, adverse event

^a^This subgroup included Id plus other chemotherapeutics or monoclonal antibody: 11 cases with doxorubicin, 7 with cyclophosphamide, 3 with thalidomide and 1 with daratumumab

Among the 13 patients who had AEs listed as one of the reasons for drug discontinuation, 9 patients endured at least one grade 3/4 AEs, including 4 who had infection, 3 who had diarrhea, 1 who had thrombocytopenia, and 1 who had abdominal distention.

## Discussion

This multi-center, retrospective, observational study highlights the real-world efficacy, feasibility, and tolerability of ixazomib-based frontline therapy in patients with NDMM.

Importantly, the results of this analysis are comparable with those of the phase 1/2 clinical trial of IRd in NDMM (NCT01217957) [12, 13].

Findings from our real-world cohort reflected the outcome of a broader general patient population, which was older than that included in the phase 1/2 trial (NCT01217957) [12, 13] (median age 67 versus 66 years), and tended to have more advanced stage diseases (ISS II–III 74% versus 57%, *p* = 0.000) and worse ECOG performance status (ECOG PS > 2 25% versus 0%, *p* = 0.000) (Table 5). Near half of patients (45%) received IRd regimen as in the phase 1/2 clinical (NCT01217957); about 30% of patients received doublet regimen of ixazomib plus dexamethasone, mostly the elderly (median age 75 years) who might not be able to tolerate triplet regimens; the rest of patients (25%) received triplet regimen of Id plus other chemotherapeutics or monoclonal antibody. Yet despite these differences in baseline characteristics and treatment exposure, treatment response and survival outcomes in our study were comparable with the trial (NCT01217957) findings, with an ORR of 95%, ≥ VGPR rate of 66%, and a 12-month PFS of 86% in our cohort compared with 88%, 58%, and 88% in the phase 1/2 trial (NCT01217957) [12, 13], respectively (Table 5). Besides, there was no statistically significant difference in treatment response between different ixazomib-based regimens. The results suggest that the ixazomib-based triplet and doublet combination as frontline therapy could be active in a broader real-world population. Of note, despite the promising efficacy results in our whole cohort, we still observed a relatively inferior treatment outcome in those who have high-risk CA and those who were ineligible for clinical trial. Although more than 86% of the patients with high-risk CA achieved disease response, only one-third achieved a deeper response of ≥ VGPR (Table 3). Besides, an inferior PFS was observed in the patients with high-risk CA and those who were ineligible for clinical trial (Fig. 2 B, F), with a 12-month PFS of 76.6% and 77.4%, respectively, which was lower than the data reported in the NCT01217957 trial (88%) [12, 13]. The possible reasons for these discrepancies may lie in the fact that the patients ineligible for clinical trial were older, with worse ECOG performance status and lower transplantation rate. In fact, patients with age more than 75 years and ECOG PS > 2 did demonstrate inferior PFS in our cohort (Fig. 2 D, E).

**Table 5:** Comparison of clinical features and outcomes data from the present study and from the phase 1/2 trial (NCT01217957) of IRd treating newly diagnosed multiple myeloma

	Present study (*N* = 85)	NCT01217957 trial (*n* = 65)	*P*
Clinical features of patients			
Median age (range)	67 (35–87)	66 (34–86)	
Age ≥ 65 years, *n* (%)	44 (52)	34 (52)	0.947
Age ≥ 75 years, *n* (%)	18 (21)	12 (18)	0.680
ISS stage at diagnosis, *n* (%)			0.000
I	22 (26)	28 (43)	
II	26 (31)	28 (43)	
III	37 (43)	9 (14)	
ECOG performance status, *n* (%)			0.000
> 2	21 (25)	0	
Effectiveness/efficacy			
ORR (≥ PR), *n* (%)	81 (95)	56 (88)	0.049
≥ VGPR, *n* (%)	56 (66)	37 (58)	0.263
12-month PFS, %	86	88	
Safety profile			
Any grade ≥ 3 AE	25 (29)	49 (75)	0.000
AE leading to discontinuation of ixazomib	12 (14)	5 (8)	0.219

*IRd*, ixazomib plus lenalidomide and dexamethasone; *ISS*, International Staging System; *ORR*, overall response rate; *VGPR*, very good partial response; *PFS*, progression-free survival; *AE*, adverse event

Safety profile was compatible with the previously published phase 1/2 trial (NCT01217957) [12, 13]. Grade ≥ 3 AEs were reported in 29% of the present cohort within follow-up, and 14% discontinued ixazomib due to AEs in the absence of disease progression. However, the lower grade ≥ 3 AE rate (29%) reported here is likely to be attributed to a reporting bias due to the retrospective nature of this study. Despite the potential bias, these findings still reflected that ixazomib-based regimens are well-tolerated. Of note, no grade > 2 PN was observed in our cohort. This finding was encouraging, especially in the consideration that PN has been one of the major concerns when using proteasome inhibitor and the reported incidence of bortezomib-induced PN was 40% [25]. Despite the lower > 3 AE rate and high response rate in our cohort, we observed high discontinuation rate (14% vs. 8%) in our data. Unlike in strictly controlled and well-managed clinical trials, the treatment endurance may be rather low in real-life practice, since physicians may be less motivated to encourage their patients on the need to continue treatment in routine practice, and patients in real-life may tend to give up a therapy despite few side effects.

In our real-world cohort, ten patients received single-agent ixazomib maintenance after induction. None of the patients discontinued ixazomib maintenance due to AEs, and all but the one who had disease progression remained on maintenance therapy until the date of the last follow-up. The favorable tolerability observed in our cohort highlighted the feasibility of maintenance therapy with ixazomib. The SCT rate in our cohort was only 15.4% in patients who were evaluated as transplantation-eligible (ages 18–64 years, ECOG 0–1, without severe comorbidities). This data was quite consistent with the reported SCT rate (14.4%) in another large real-world study of 940 Chinese NDMM patients [26], and it did reflect the real situation in China. The possible reasons for the low SCT rates include fears for transplant, financial concerns (many fees for SCT, including melphalan, were not covered by health insurance), limited drug accessibility (melphalan has just commercially entered China in 2019), shortage of qualified medical service (only large reference centers or specialized centers have the ability to perform SCT), and disturbance on routine medical service during COVID.

The ixazomib-based all-oral regimens, including IRd, ICd, and ITd, have demonstrated promising efficacy, safety, and tolerability in rigorously controlled clinical trials for NDMM [12–15, 27, 28]. Besides the greater convenience brought by the oral administration, the NDMM patients who received all-oral regimens have been shown to have lower economic burden of illness, less activity impairment, and lower productivity loss [29]. These facts together explained the reason for choosing ixazomib-based regimens as frontline therapy in selected real-world patients. More importantly, all-oral therapy may be able to help in decreasing the exposure to and infection with COVID-19 [16].

Indeed, real-world data are emerging for the use of ixazomib in RRMM, but such data in the frontline treatment setting with ixazomib are still limited. The findings in the present study are encouraging. Although the follow-up was fairly short (median 10.3 months), as ixazomib was just introduced in China in April 2018, our real-world data did demonstrate that the ixazomib-based regimens were active in routine practice for NDMM. On the basis of this analysis and the other co-factors that should be taken into consideration when making treatment decisions NDMM patients in routine practice, ixazomib-based regimen would be an optimal alternative as frontline therapy.

In conclusion, herein we reported the data from the first real-life, multi-center study on the effectiveness and safety profile of ixazomib-based frontline therapy in NDMM.

### Authorship contributions

J.L., L.B., Z.-J.X, S.-L.W., X.Z., K.-Y.D., W.-H.Z., W.Y., B.-Z.L, C.-C.F., B.C., L.-M.H., L.W., J.L., Y.Y., T.-H.X., W.-D.W., Y.H., and G.-L.W. collected data; J.L. analyzed results and created the figures; L.B., Z.-J.X, and P.L. designed the research; J.L. drafted the manuscript; and L.B., Z.-J.X, and P.L. gave the final approval of the paper.

## Data Availability

The data that support the findings of this study are available from the corresponding author upon reasonable request.
